# The interplay between cerebellum and basal ganglia in motor adaptation: A modeling study

**DOI:** 10.1371/journal.pone.0214926

**Published:** 2019-04-12

**Authors:** Dmitrii I. Todorov, Robert A. Capps, William H. Barnett, Elizaveta M. Latash, Taegyo Kim, Khaldoun C. Hamade, Sergey N. Markin, Ilya A. Rybak, Yaroslav I. Molkov

**Affiliations:** 1 Department of Mathematics and Statistics, Georgia State University, Atlanta, Georgia, United States of America; 2 Department of Neurobiology and Anatomy, Drexel University College of Medicine, Philadelphia, Pennsylvania, United States of America; 3 Neuroscience Institute, Georgia State University, Atlanta, Georgia, United States of America; University of Haifa, ISRAEL

## Abstract

Motor adaptation to perturbations is provided by learning mechanisms operating in the cerebellum and basal ganglia. The cerebellum normally performs motor adaptation through supervised learning using information about movement error provided by visual feedback. However, if visual feedback is critically distorted, the system may disengage cerebellar error-based learning and switch to reinforcement learning mechanisms mediated by basal ganglia. Yet, the exact conditions and mechanisms of cerebellum and basal ganglia involvement in motor adaptation remain unknown. We use mathematical modeling to simulate control of planar reaching movements that relies on both error-based and non-error-based learning mechanisms. We show that for learning to be efficient only one of these mechanisms should be active at a time. We suggest that switching between the mechanisms is provided by a special circuit that effectively suppresses the learning process in one structure and enables it in the other. To do so, this circuit modulates learning rate in the cerebellum and dopamine release in basal ganglia depending on error-based learning efficiency. We use the model to explain and interpret experimental data on error- and non-error-based motor adaptation under different conditions.

## Introduction

Motor learning is a process of acquiring skills to perform an appropriate motor task in response to a sensory cue, e.g. precise reaching with a mouse pointer to a target spot shown on the screen. Motor adaptation is a form of motor learning to overcome movement perturbations caused by novel environment or altered sensory feedback. During motor adaptation, future movements are corrected using error information acquired on previous trials [1]. Representation of the movement error depends on available sensory components. For example, during reaching movements under unexpected perturbation, visual feedback can provide a vector displacement of the movement endpoint relative to the target position. This vector error may be used by the central nervous system to adjust motoneuron activity and eliminate the effects of the perturbed environment. It has been suggested that this process involves the cerebellum that adjusts the internal model of the body based on information about movement error [2]. This type of motor adaptation is often referred to as supervised or error-based learning [3].

Error-based learning fully relies on availability and correct representation of the movement error. However, in certain conditions the necessary information about error is limited or unavailable. Several related experimental conditions were proposed. For example, in reaching experiments, the subject’s arm can be covered with a non-transparent screen and visual feedback is transformed by rotating the image of the movement on a computer screen [4], inverting the image with a Dove prism [5], etc. When adequate visual feedback is not available, error-based learning becomes impossible. However, motor adaptation can still be observed if some other measure of the movement inaccuracy is provided. This may include a score representing the distance from the movement endpoint to the target, or a reward for a reach performed within a target spot [6, 7]. This type of motor adaptation is referred to as non-error-based learning and is mediated by the basal ganglia (BG) via reinforcement of more successful movement attempts [2, 3].

The emergent view is that motor adaptation can be simultaneously provided by two distinct learning mechanisms: (1) an error-based learning mechanism performed by the cerebellum and (2) a non-error-based learning mechanism that operates in BG. Importantly, these two learning mechanisms differ in their use of sensory information (vision, proprioception, etc.). In the error-based (supervised) learning mechanism, sensory input is explicitly used to compute a vector displacement between the final position and a given target position, and the magnitude of this displacement is reduced with every trial. In contrast, the non-error-based (reinforcement) learning mechanism does not explicitly calculate the distance to a target and relies on whether the “reward” value for the performed movement is better than for the previous ones.

Various experimental setups were implemented to experimentally distinguish between error based and non-error-based learning. The most intriguing results were obtained in experiments where visual feedback was so distorted that efficient error-based learning was no longer possible. For example, Gutierrez-Garralda et al. [5] used a Dove prism as a visual perturbation while the participants attempted to throw a ball at a target, so that their perceived error was inverted. In these conditions, faulty cerebellar error-based learning would cause participants to perform progressively worse at the task. The control group of participants was still able to adapt to the perturbation, while the groups with impaired basal ganglia function failed to adapt. Interestingly, in experiments where instead of a Dove prism, a shifting wedge prism (that did not change perception of the displacement) was used, participants adapted to the perturbation regardless of the basal ganglia integrity. Because of this, Gutierrez-Garralda et al. [5] suggested that participants suppressed malfunctioning error-based learning mechanisms and engaged the BG-based reinforcement learning to successfully adapt to the visual distortion used. However, it remains unclear what triggered one of the two mechanisms to operate or disengage depending on conditions.

Previously, we suggested a mathematical model of reinforcement-based motor adaptation [8] for planar reaching movements [9]. The objective of the present study was to explain how supervised or reinforcement learning could be engaged or suppressed during motor adaptation depending on the perturbation. To do so we augmented the model by including a cerebellar compartment performing trial-to-trial movement correction based on supervised learning. Using the extended model, we suggest a simple intrinsic mechanism that may orchestrate the involvement of error- or non-error-based learning via regulating the cerebellar learning rate and dopaminergic signaling in basal ganglia.

## Results

In this study, we modeled the neuromechanical control of reaching movements in humans and used this model to reproduce and explain the results of previous experimental studies demonstrating motor adaptation during reaching [7, 10]. In these experiments the participants did not have direct visual feedback from the arm. Instead, the arm endpoint was represented as a cursor on a display. During each experiment, different targets appeared on the screen and the task was to move the arm so that the cursor would reach the target. Such a setup allowed an experimenter to easily alter the visual feedback if needed. The applied perturbations included image rotations around the movement starting point and reflection across to the vertical axis. In presence of the perturbation, the participants had to learn to reach the target relying on the distorted visual feedback or other available information. To prevent the subjects from making correction during the movement, they were either forced to perform movements as quickly as possible or the cursor was only shown on the screen when the movement was complete.

Previously we developed a model for neural control of goal-directed reaching movements that simulated the entire pathway from the motor cortex through spinal cord circuits to the muscles controlling arm movements [9]. In that model, the arm consisted of two joints (shoulder and elbow), whose movements were actuated by six muscles (4 single-joint and 2 two-joint flexors and extensors). Cortical inputs were calculated by a cortical “controller” based on (a) an internal model of the arm, (b) a proposed straight-line trajectory to a target position, and (c) a predefined bell-shaped velocity profile. The neural controller generated a motor program (six time-varying signals) producing a task-specific activation of low-level spinal circuits that in turn induced the muscle activation pattern resulted in the intended reaching movement. In our present study, the internal model of the body used to calculate the motor program, or the perception of the target position could be perturbed, resulting in an imprecise or completely faulty reaching movement. Therefore, we augment the model by sequentially adding structures responsible for different forms of motor learning allowing us to reproduce the experimental data on motor adaptation to the applied perturbations.

The overall organization of the Results section is as follows. First, we incorporate the error-based learning mechanism performed by the cerebellum, in the model. The mechanism modifies the motor program based on supervised learning. Then, we show that if the perception of movement error is strongly distorted, the error-based adaptation quickly worsens the performance. Experimentally, it was found that the subjects switch to a different non-error-based mechanism of motor adaptation mediated by BG in the latter situation [5]. Following our previous publication [8], we add the BG network to the model which participates in forming the cortical motor program based on reinforcement of previously successful reaching attempts or on suppression of unsuccessful ones. Via simulations we show that if not orchestrated, the supervised and reinforcement learning processes do not get along. To avoid their interference, we incorporate a simple mechanism which regulates the learning rate in cerebellum and dopaminergic signaling in striatum and show its efficiency in endogenous switching between error-based and non-error-based learning mechanisms. Finally, we compare our model performance with existing literature data on the subject.

### Supervised learning in cerebellum during reaching

**Fig 1:**
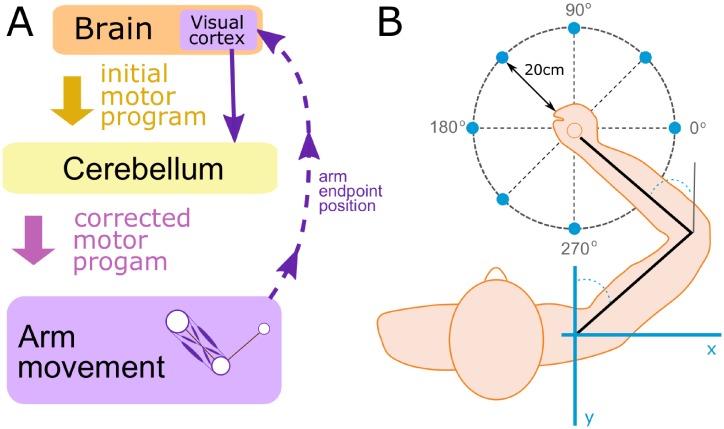
General model structure. **(A)** To perform a reaching movement the brain creates a motor program and sends it as an input to the cerebellum implemented as an artificial neural network. The cerebellum modifies the motor program and sends it as an input to the neuromechanical arm model. The perceived displacement between the movement endpoint and the target position (the vector error) is fed back to the cerebellum (visual feedback) and used to calculate the adjustment of weights in the cerebellar network using error back propagation. **(B)** We simulated reaching movements which started from a fixed initial position, aiming to reach a target located on a circle centered at the starting point with a radius of 20 cm. The direction of the movement relative to the body position was defined by the angle as shown.

In the present model we used the previous model [9] as a basis, but added a model of cerebellum as an artificial neural network with an input from the cortical controller and the output to the spinal cord network (Fig 1), whose function was to alter the motor program in order to correct the movement. The synaptic weights of the cerebellar network were updated based on a classical error back propagation procedure [11] using a squared distance between the movement endpoint and the target (the squared error) as a cost function (see Methods for details). Simply said, if the produced movement failed to precisely reach the target, the model adjusted the weights in the cerebellar network proportionally to the derivatives of the squared error with respect to the weights. The coefficient of proportionality is called the *learning rate* which defines how quickly the system adapts to the perturbation. The model also accounted for a trial-to-trial *degradation* of the cerebellar network weights which reflected “forgetting” process in the system and defined how quickly the accumulated correction would wash out if visual feedback was disrupted.

To illustrate the process of error-based motor adaptation provided by the cerebellum, we simulated reaching movements in conditions of two different visual perturbations. Each simulation consisted of three consecutive phases. During the first phase, we simulated reaching to a target located in 90 degrees direction (see Fig 1B for definition of directions) with veridical feedback (i.e. the perceived endpoint position and expectation of where it should appear as a result of the movement coincided). During the second phase, a visual perturbation was introduced. Here we used two different perturbations: a *shift* and a *rotation*. The rotation perturbation mimicked the rotation of the image around the initial movement point by 30 degrees counterclockwise so that the perceived target position was at 120 degrees (see Fig 1B). Note, that during such a perturbation the perceived vector error (the perceived displacement of the movement endpoint relative to the target) is rotated by the same angle relative to the actual vector error. The shift perturbation consisted in parallel translation of the image in such a way that the target was perceived at the same location as during the rotation perturbation. The difference was in perception of the vector error which was not perturbed during the shift perturbation. During the third phase, the veridical feedback was restored.

**Fig 2:**
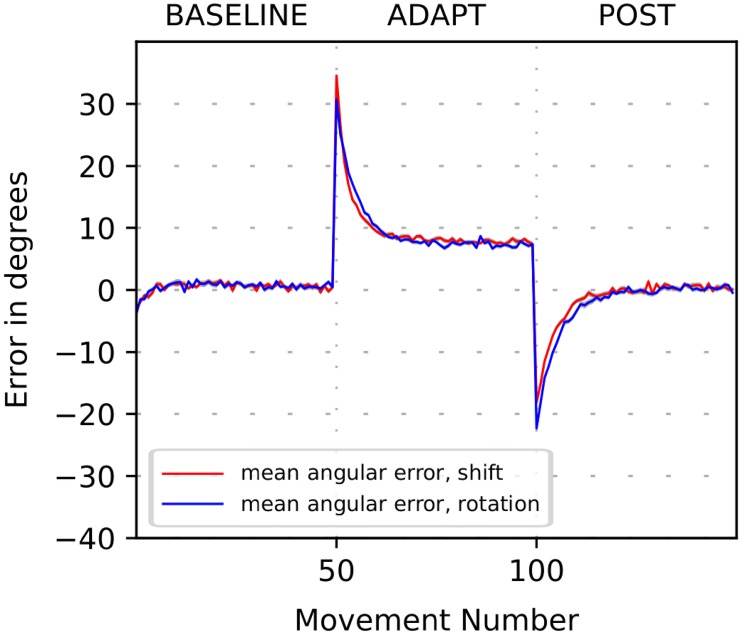
Simulation of cerebellar-based adaptation to mild perturbations. The plot shows the movement error in degrees versus trial number during simulation of the 3-phase experiment when a visuomotor perturbation is introduced in the beginning of ADAPT phase and removed in the beginning of POST phase. The perturbations are the shift (red line) and 30-deg visuomotor rotation (blue line) (see text for details). Solid lines show the 32-run per trial average of the angle difference between the perceived target position and the perceived hand/cursor position versus the trial number. After the perturbation is introduced, the initial error is approximately equal to the shift/rotation angle. Then it converges to the asymptote below 10 degrees. The asymptote is nonzero due to “forgetting” effect presented in error-based learning, corresponding to the damping term in the cerebellum model. After the perturbation is removed, the error changes its sign and abruptly increases in magnitude again (perturbation aftereffect). Then it converges back to zero.

Fig 2 depicts motor adaptation results to these two perturbations as generated by the model. During the first “BASELINE” phase the model generated movements that reached closely to the target within natural movement variability (see Methods for details). After perturbation (see ADAPT in Fig 2), the model missed the target by the angle which initially coincided with the magnitude of the perturbation of 30 degrees, but then the angular error gradually reduced to below 10 degrees on subsequent trials as the cerebellum learned to correct the movement. When the perturbation was removed (see POST in Fig 2), the perceived and actual target positions coincided again. However, at the beginning of the POST phase, the cerebellum network was primed to counteract the previous perturbation, so it required certain number of trials for the system to come back to baseline. This phenomenon is often referred to as an *aftereffect* of a perturbation.

Incomplete elimination of errors, observed in the end of ADAPT epoch of Fig 2 is well-known effect [12], widely observed in experimental studies of motor learning. To our knowledge so far there is no theory for the neural origin of it. Phenomenologically it is usually interpreted as a result of the balance between learning and “forgetting”. In a linear Kalman-filter like state space model of the CB it is implemented by adding a damping term to the equation. Our model also has this damping term (which we call “degradation”) that leads the error to saturate at a non-zero level defined by the balance between learning and degradation processes. See Methods section for implementation details.

As mentioned above, the rotation perturbs the perception of movement error and thus may adversely affect the supervised learning process relative to the shift. Interestingly, for a perturbation of the magnitude used, the courses of adaptation to the shift and the rotation were very similar. Thus, 30 degrees rotation did not distort the vector error to noticeably impair the error-based motor adaptation.

### Model calibration

We used the experimental protocol and data from the study of Schlerf et al. [10] to calibrate the model parameters (see Schlerf et al. context in Methods), and reproduce some of their results. In short, in their experiments, the participants performed slicing movements to reach the targets appearing on the screen at a 10 cm distance from the starting location. The directions to the targets were uniformly randomly distributed within the sector from 75–15 to 75+15 degrees (see Fig 1B for directions). The participants were divided in two groups: healthy controls and cerebellar degeneration patients.

First, the participants had to adapt to visuomotor rotation introduced in 5-degree steps. There were 4 incremental steps with successive rotations by 5, 10, 15, and 20 deg. The 20-deg rotation was then repeated and followed by 5-deg decrements involving rotations by 15, 10, and 5 deg. Each step involved 16 trials. Next, there was an alternation between blocks of trials with no rotation and blocks of trials with an imposed 20-deg rotation. The rotation was introduced twice, with each block sandwiched between no-rotation blocks.

**Fig 3:**
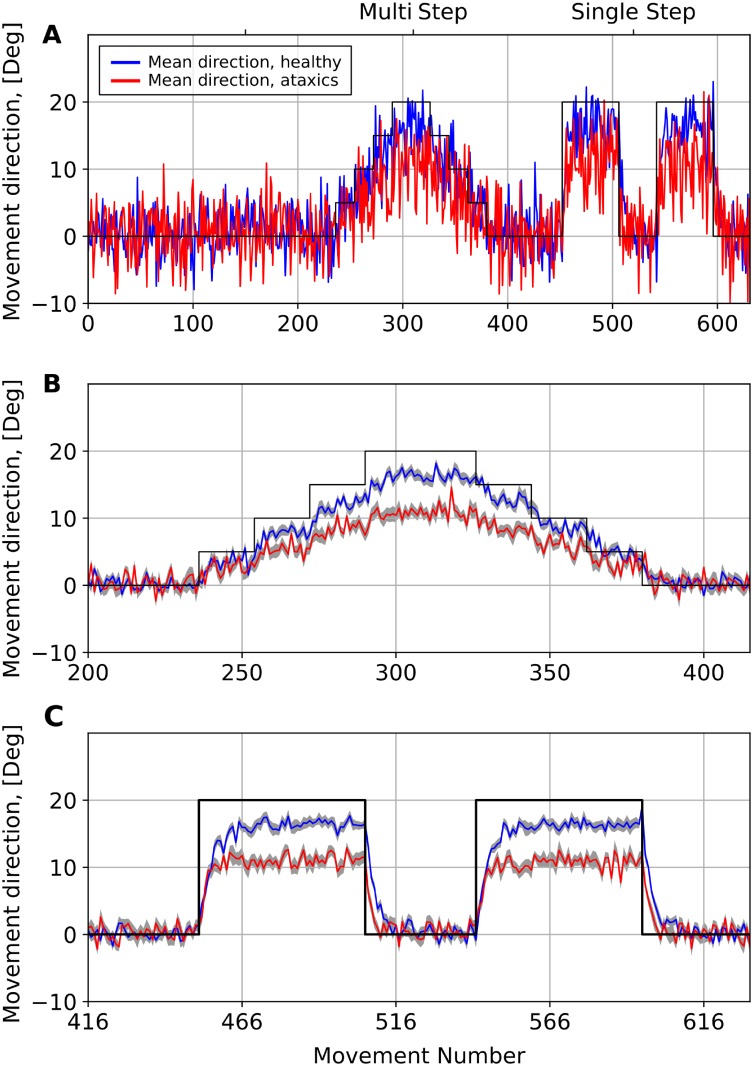
Simulations of adaptation to the visuomotor rotation perturbations. Each plot shows reaching angle dynamics which is defined here as a difference between the perceived target position and the movement direction. In this simulation (similar to Schlerf et al. [10] experiments) a visuomotor rotation first was introduced and removed in a stepwise manner (Multi Step phase), then it was introduced and removed abruptly (Single Step phase). The solid black line shows the actual target position relative to the perceived target position (rotation angle); the blue line shows simulation corresponding to the control group in Schlerf et al.; the red line shows the simulation with increased degradation rate and increased noise in the movement endpoint to mimic the cerebellar ataxia group data from Schlerf et al. (see text for details); the light grey areas show the standard error of the mean (SEM) over 16 runs. (**A**) one trial simulation of the control (blue) and ataxic (red) conditions; (**B**) 16-runs average multistep perturbation adaptation close-up (**C**) 16-runs average single step perturbation adaptation close-up.

We used these data to calibrate the model by tuning the learning rate, the degradation rate and the movement endpoint variability magnitude in both control and cerebellar patient groups to fit the adaptation times and asymptotic errors in all cases. Fig 3 represents our simulations which are remarkably similar to the experimental data in Fig 2 from [10]. Fig 3A shows simulations of 650 trials (reaching movements) as deviations of movement directions from the unperturbed target. The solid black line shows the perturbation angle which changes from 0 to 20 deg and back again in stepwise manner between trials 200 and 400, and abruptly between trials 450 and 600. In the model, after calculating the movement endpoint, we add to it a random number with standard deviation characterizing the magnitude of uncontrolled movement variability. This results in a significant noise in movement direction as seen in Fig 3A. In Fig 3B and 3C to reduce the noise we show the averages over 16 runs for the perturbation epochs only. In this representation, one can clearly see the adaptation process with subsequent saturation of the error at a certain level (the asymptotic error, see Fig 3C).

The saturation of the error happens when balance is achieved between the learning and degradation processes, i.e. when the change in weights due to learning in the cerebellar network is precisely compensated by their decay. Therefore, the asymptotic error is a decreasing function of the learning rate and an increasing function of the degradation rate. In contrast, the adaptation time (time to saturation) is decreasing with both rates which makes it possible to infer the learning rate and the degradation rate from Schlerf et al. data with high accuracy.

In study of Schlerf et al. [10] there was a striking difference between the asymptotic errors in the control group and cerebellar ataxia patients (see Fig 3C for our simulations reproducing the same). As explained above, greater asymptotic errors can result from the lower learning rate or from faster degradation. Schlerf et al. used a simple Kalman filter model to analyze the experimental data which showed that patients with cerebellar degeneration had slightly higher learning rate and significantly shorter memory corresponding to the higher degradation rate. Besides, the variability of movement endpoint in cerebellar patients was significantly larger than in healthy controls. Our results are largely consistent with Schlerf et al. conclusions. Specifically, the movement endpoint noise magnitude (motor noise hereinafter) was increased to replicate the larger movement variability observed in ataxic patients. Motor noise magnitude *per se* hardly influenced the adaptation efficiency (not shown). To reproduce data from ataxic patients, we have increased the cerebellar degradation rate, which allowed the model to reproduce the asymptotic errors in this group (Fig 3).

### Cerebellum model fails to adapt to strong perturbations

**Fig 4:**
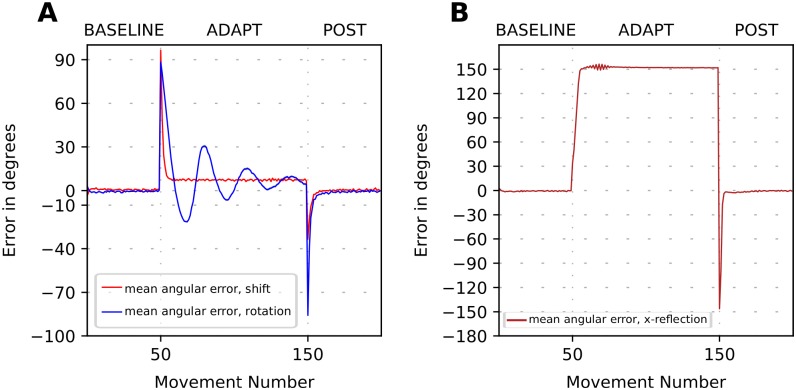
(Lack of) Error-based adaptation to strong visuomotor perturbation. The plot shows movement error in degrees versus trial number during simulation of the 3-phase experiment when a visuomotor perturbation is introduced in the beginning of ADAPT phase and removed in the beginning of POST phase. Solid lines show the 32-run average angle difference between the perceived target position and the perceived hand/cursor position. (**A**) Simulation of adaptation to 90 degrees visuomotor rotation (blue line) and 90 degrees shift (red line). (**B**) Simulation of adaptation to the x-reflection perturbation. Note that the perturbation size is the same for the shift and rotation perturbation in (A). However, the trial-to-trial dynamics of the error are very different, as well as the magnitudes of the aftereffects.

After calibration, the model presented above provides motor adaptation closely reproducing experimental data in the context of mild visual perturbations. However, as we describe below, it failed to adapt to strong visual perception perturbations. As examples of such perturbations we simulated the rotation by 90 degrees and reflection of the visual field in horizontal direction (see Fig 4). Both perturbations not only required the model to overcome a significant distance between the expected and perceived cursor location but should also deal with strong distortions of the vector error.

Reflection perturbations were previously used in throwing experiments [5] where a dove prism was used to horizontally reverse the visual field and shift the target’s position. Under such a visual perturbation, the sign of x-coordinate of the vector error is reversed, and therefore when a participant misses the actual target to the right, she observes the displacement of the movement to the left of the perceived target. In case of error-based motor adaptation the correction should consist in throwing further to the right, thereby increasing the magnitude of the error distance trial-to-trial. As this did not happen in their experiments, the authors of [5] concluded that error-based learning was not possible.

We simulated the model of cerebellar correction in strong rotation and reflection contexts (see Methods for details). The simulation results in Fig 4 clearly show that the model performance was much worse when the vector error was significantly perturbed compared to mild perturbations. For the 90 deg rotation, the model produced oscillations instead of gradually converging to the target (compare the shift and rotation cases in Fig 4A). This behavior occurred because the *x* and *y* coordinates of the vector error received by CB were transformed by the rotation in such a way, that a flawed correction produced by the cerebellum model did not make the next reaching endpoint to become closer to the target.

During the reflection perturbation, the cerebellar corrections became so faulty that the model diverged from the desired target (Fig 4B). This happened because the sign of the *x* coordinate of the vector error was inversed. Thus, each adjustment of the cerebellar network weights shifted the reaching endpoint along the *x* axis in the direction opposite to the desired one. Additionally, since the error magnitude increased for each trial, these faulty corrections became progressively stronger on consecutive trials. In the simulation shown in Fig 4B, the direction of the movement diverges from the target.

**Fig 5:**
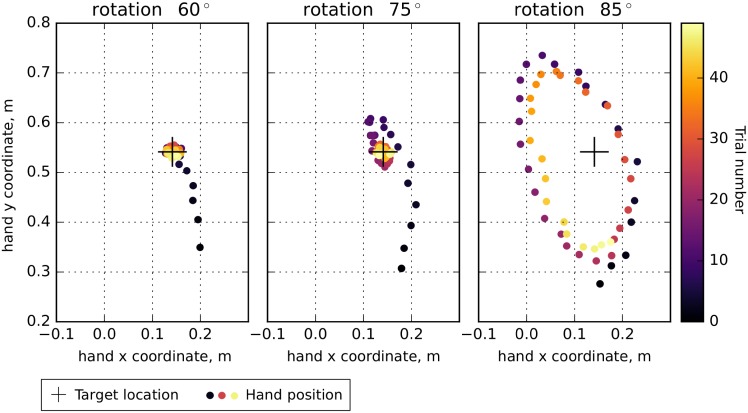
Adaptation to visuomotor rotation with increasing angle. The panels show successive movement endpoints obtained from simulation during adaptation to rotation by 60, 75 and 85 degrees. The color of each point represents the trial number. The ‘+’ shows the target position. Only the adaptation phases of individual simulations are plotted. To emphasize the effect, the degradation rate in the cerebellum was set to zero. As the rotation angle increases, the trajectory first starts spiraling while converging to the target (the middle panel) and then exhibits oscillations around the target with non-decaying amplitude (right panel).

Fig 5 illustrates that the performance of the cerebellum model deteriorates continuously with the increase of the perturbation magnitude. In these simulations, we varied the angle of visual rotation and observed the resulting changes in the model’s performance. In Fig 5, we show the adaptation phase dynamics. The model was able to successfully adapt to rotations as large as 60 degrees. However, for stronger rotations, the trajectory of the movement endpoint during adaptation started to curl as the vector error components became increasingly distorted by the rotation, as the rotation angle increased. For moderate rotation angles (see 75 deg rotation in Fig 5) the model movement still converged to the desired target in a manner known in ODE theory as “stable focus”. However, for rotation angles greater than 80 deg, the convergence to the target no longer happened, and the endpoint trajectory started to spiral around the target (Fig 5C) with progressively larger amplitude as the rotation angle increased.

### Combining error-based and non-error-based mechanisms

**Fig 6:**
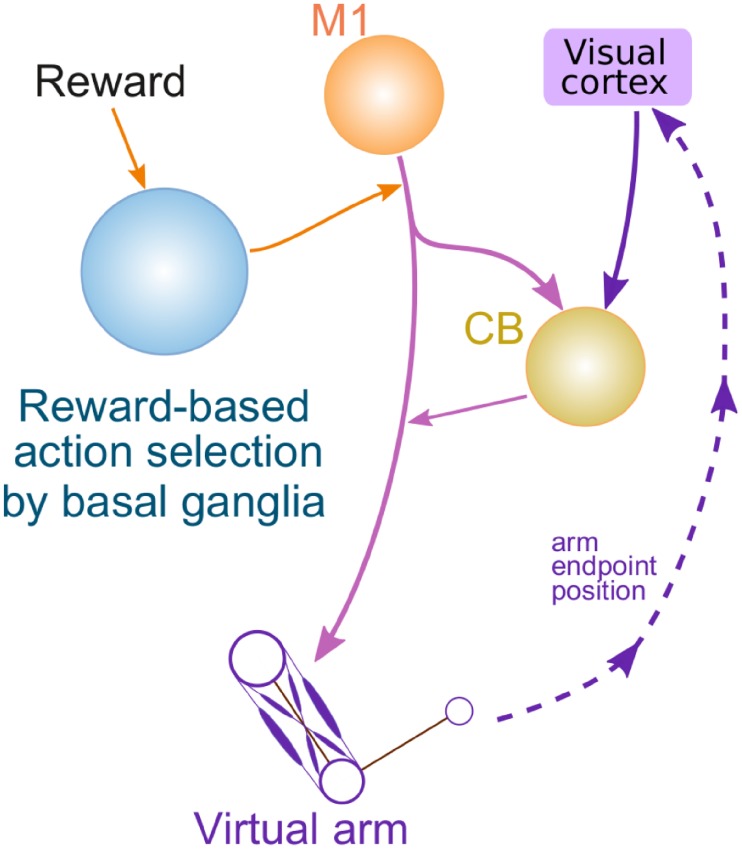
General architecture of the model with cerebellum and basal ganglia. In this version of the model basal ganglia select a motor program for execution among possible behaviors generated in the motor cortex (M1). The cerebellum (CB) receives a copy of the motor program and modifies it before the result is fed to the movement system.

In the previous section, the supervised/error-based learning performed by the model of cerebellum was the only motor adaptation mechanism. As we noted before, it was previously suggested that when error-based learning becomes impossible (e.g. in case of strong visual rotations/reflections, see above), reinforcement/non-error-based learning mechanisms presumably operating in BG are engaged. Thus, we added a model of BG that was responsible for action selection and reinforcement (Fig 6). In this model architecture, BG are involved in action selection hence contribute to formation of the motor program sent downstream in response to a visual cue representing the target position for reaching movements (Fig 6, see [8] for details).

After the movement was complete, the trial performance was evaluated based on how close the movement endpoint was to the target which defined the reward amount. Depending on experimental settings, the reward can be explicitly defined (e.g. fixed reward amount was provided in the case when reaching ended within the target spot) or represented as a measure of satisfaction by the results of movement performed. In the latter case, to calculate the reward amount we used a score monotonically decreasing with the distance from the movement endpoint to the target position (see Methods for details).

The action selection process in BG was modulated by the reinforcement learning procedure. Reinforcement or suppression of the selected actions depended on the sign (and magnitude) of the reward prediction error (RPE) which was implemented as a temporal difference between the rewards obtained on the current and previous trials (see [8] for details). Positive RPE indicated that performance improved on the current trial, and, therefore, the selected action was reinforced. Negative sign of the RPE indicated that the performance worsened, so future selection of this action was suppressed which triggered exploration process for more rewarding actions [8].

#### Exploration due to negative reinforcement disables error-based motor adaptation in case of mild perturbations

**Fig 7:**
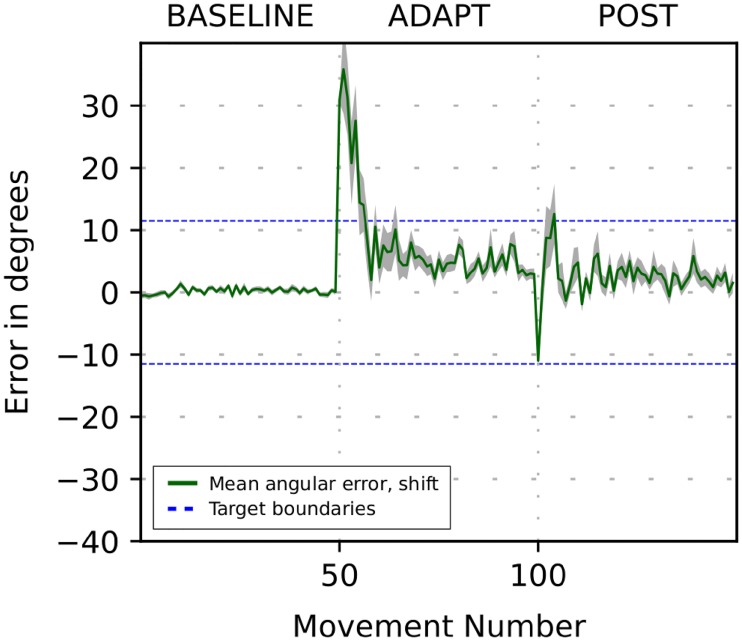
Adaptation to a mild perturbation in the model with cerebellum and basal ganglia. The plot shows error magnitude versus trial number during simulation of the 3-phase experiment when a visuomotor perturbation is introduced in the beginning of ADAPT phase and removed in the beginning of POST phase. Solid line shows 32-runs average angle difference between perceived target position and perceived hand/cursor position. Grey area shows SEMs. Blue dashed lines show the rewarding range.

Using the augmented model, we simulated the mild perturbation contexts with the reward provided if the reaching endpoint is within a fixed distance from the target. We found (see Fig 7) that after addition of BG, the model could adapt to a visual shift and mild rotation (not shown), but the time course of this adaptation was different (compare to Fig 4). When the perturbation was introduced at trial 50 (Fig 7), the model missed the target, and the reward was not provided. This resulted in a negative reward prediction error (RPE) which led to a negative reinforcement of the previously performed movement. The BG network suppressed the selection of that movement and initiated the exploration process (see increased variability of the errors between trials 40 and 50 in Fig 7). Eventually, the model generated a reaching movement with an endpoint within the rewarding spot. The provided reward resulted in a positive RPE which reinforced selection of the same movement on the subsequent trials.

Note that during exploration process, the CB network continued to generate corrections of the motor program to reduce the movement error on subsequent trials expecting that the same cortical input would be provided. However, these corrections were not efficient as a different action was selected on every trial during exploration. During the adaptation, the accumulated change in the CB network synaptic weights appeared to be insignificant which was evident from the very weak aftereffect as the perturbation was removed after trial 100 in Fig 7.

#### Faulty CB corrections destroy reinforcement-based adaptation in case of strong perturbations

**Fig 8:**
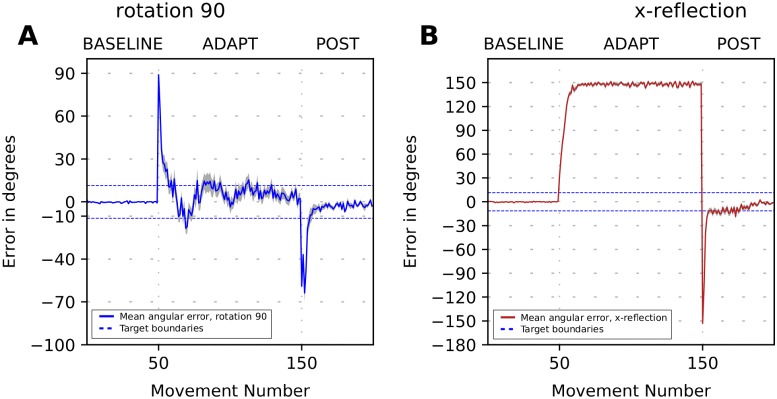
Adaptation to strong perturbations in the model with cerebellum and basal ganglia. The plot shows error magnitude versus trial number during simulation of the 3-phase experiment when a visuomotor perturbation is introduced in the beginning of the ADAPT phase and removed in the beginning of the POST phase. The solid black line shows the 32 runs average angle difference between the perceived target position and the perceived hand/cursor position during adaptation to the 90-degree rotation (**A**) and the x-reflection (**B**) perturbations. Dashed lines show the rewarding range. Grey area shows SEMs. BG addition neither improve adaptation to the strong rotation, nor does it make the adaptation to the reflection perturbation possible.

We have shown above that when the vector movement error is distorted by a strong visual rotation or a reflection of the visual field, the error-based mechanisms lead to divergence of the movement endpoint from the target instead of adaptation to the target. Here we show that the same effect is observed in presence of reinforcement-based mechanisms, too. The simulations of the model with reinforcement and error-based mechanisms combined are shown in Fig 8.

This version of the model also produced oscillations after the rotation by 90 deg (Fig 8A). Similarly, in case of the reflection perturbation (Fig 8B), the time course of the error was virtually identical to the one produced by the model with error-based adaptation mechanism only (compare to Fig 4B). Fig 8B illustrates that the inverted *x* component of the vector error produced growing cerebellar correction that ultimately resulted in the movement in the direction opposite to the target regardless of the motor program selected by BG.

#### Context-dependent control of error-based learning

Results presented in the paragraphs above suggest that visual perturbations, which strongly distort vector error feedback (e.g. visual reflections), make motor adaptation impossible. However, this prediction contradicts the experimental evidence. Indeed, previous studies suggest that the motor control system stops relying on error-based learning mechanisms in situations when perception is strongly perturbed [5, 13]. This can be achieved if there is a mechanism that prevents the cerebellum from further adjusting the motor command given that previous adjustments did not lead to improvement in performance.

**Fig 9:**
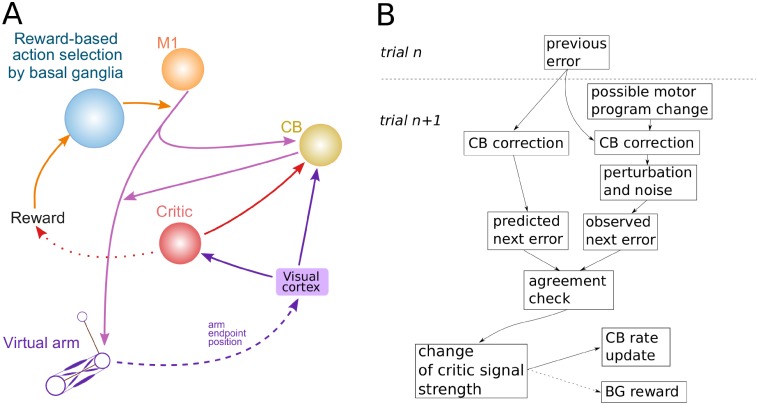
Basal ganglia and cerebellum interaction through the critic and critic mechanism. Panel A: In this version of the model **t**he critic node controls the learning rate of the cerebellum (CB) and offsets the reinforcement signal in basal ganglia. It increases or decreases its output depending on whether the predicted error after correction corresponds to the observed one or not. The critic output is used to set the CB learning rate and add to the dopamine release in striatum. In case the error-based/cerebellar correction is successful, the CB learning rate is increased on the next trial, and the striatal dopamine concentration is increased to prevent negative reinforcement of the previously selected action. In case the error-based correction failed, the CB learning rate is reduced, and the striatal dopamine concentration is not modified thus allowing for negative reinforcement. Panel B: The diagram clarifies how the critic output is computed based on the different pieces of information from the previous (n) and current (n+1) trials. After the previous trial cerebellum modifies the synaptic weights and forms a prediction about the next error value based on that modification. At the current (n+1) trial the actually observed error can differ from the one predicted by the cerebellum due to influence of the factors that cerebellum cannot control and detect directly: possible change of the motor program (e.g. because of basal ganglia activity), perceptual perturbations and noise. If the observed error agrees with the predicted one, the critic signal is increased. If it strongly disagrees, the signal is decreased. Otherwise it is left unchanged. The critic output is used to set the future CB learning rate and add to the reward prediction error when calculating the dopamine level in striatum.

As discussed in previous sections, cerebellar corrections become faulty when the vector error provided to CB is unreliable (e.g. *x*-reflection perturbations). In these situations, the CB’s error based (supervised) learning mechanism cannot assist in reaching closer to the target. When error feedback is adequate, it is possible to predict the reduction of the movement error resulting from the adjustment of the CB synaptic weights (see Methods). Therefore, a simple comparison between the predicted and actual changes of the movement error can serve as a robust indicator of the error feedback reliability. Therefore, we propose and implement the performance assessment component (the critic) that compares the predicted and actual vector error in the model (see Fig 9A). If the two are consistent (i.e. the difference between the predicted and observed errors is small), the critic increases the rate of the supervised learning in CB on subsequent trials (see Methods). If the predicted and actual error changes are inconsistent, the critic reduces the learning rate. Predicted and actual errors are considered consistent (inconsistent) if their difference is within (larger than) a certain threshold. The threshold is proportional to the amplitude of the movement endpoint noise, which is constant (in particular, it is not under the model control, see Methods for details).

**Fig 10:**
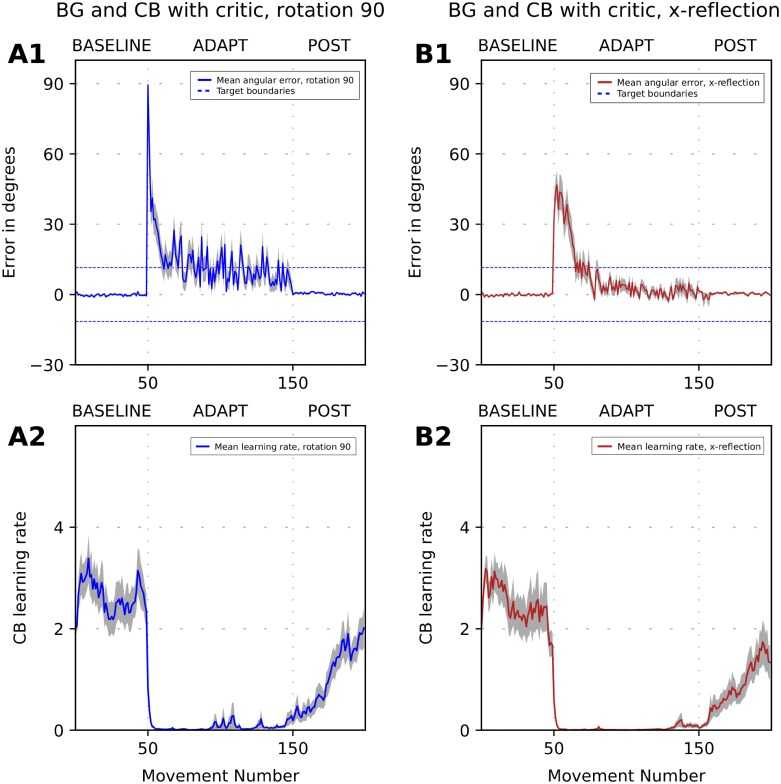
Simulation of adaptation to strong perturbation with the critic-controlled learning rate. The plot shows error size versus trial number during simulation of the 3-phase experiment when a visuomotor perturbation is introduced in the beginning of ADAPT phase and removed in the beginning of POST phase. Panels (A1), (B1) show the 32-runs average reaching error dynamics for the adaptation to 90-deg rotation (A1, blue line) and x-reflection (B1, red line). Panels (A2), (B2) show 32-runs average cerebellum (CB) learning rate dynamics for 90-deg rotation (A2, blue line) and x-reflection (B2, red line). Dashed lines show basal ganglia (BG) target size. Grey area shows SEMs. Critic control of the learning rate allows to adapt to both strong perturbations. Note absence of aftereffects.

The performance of the model for strong visual rotation and reflection of the visual field is shown in Fig 10. At the baseline, the CB learning rate fluctuated around 3 (Fig 10A2 and 10B2). During the adaptation phase, the critic immediately diagnosed that CB corrections did not produce the predicted effect and reduced the learning rate to near zero. After the perturbation was removed, the CB learning rate returned towards the baseline value.

As mentioned above, the critic detects faulty cerebellar corrections to prevent further adjustment of synaptic weights in the cerebellar network. Once the learning process is suppressed, the synaptic weights start decaying, which, in turn, gradually diminishes the accumulated faulty correction by means of the cerebellar state degradation. In Fig 10, by the end of the adaptation phase, the cerebellar correction almost fully vanished which is evident from the lack of aftereffects after the perturbation is removed.

#### Context-dependent control of reinforcement learning

**Fig 11:**
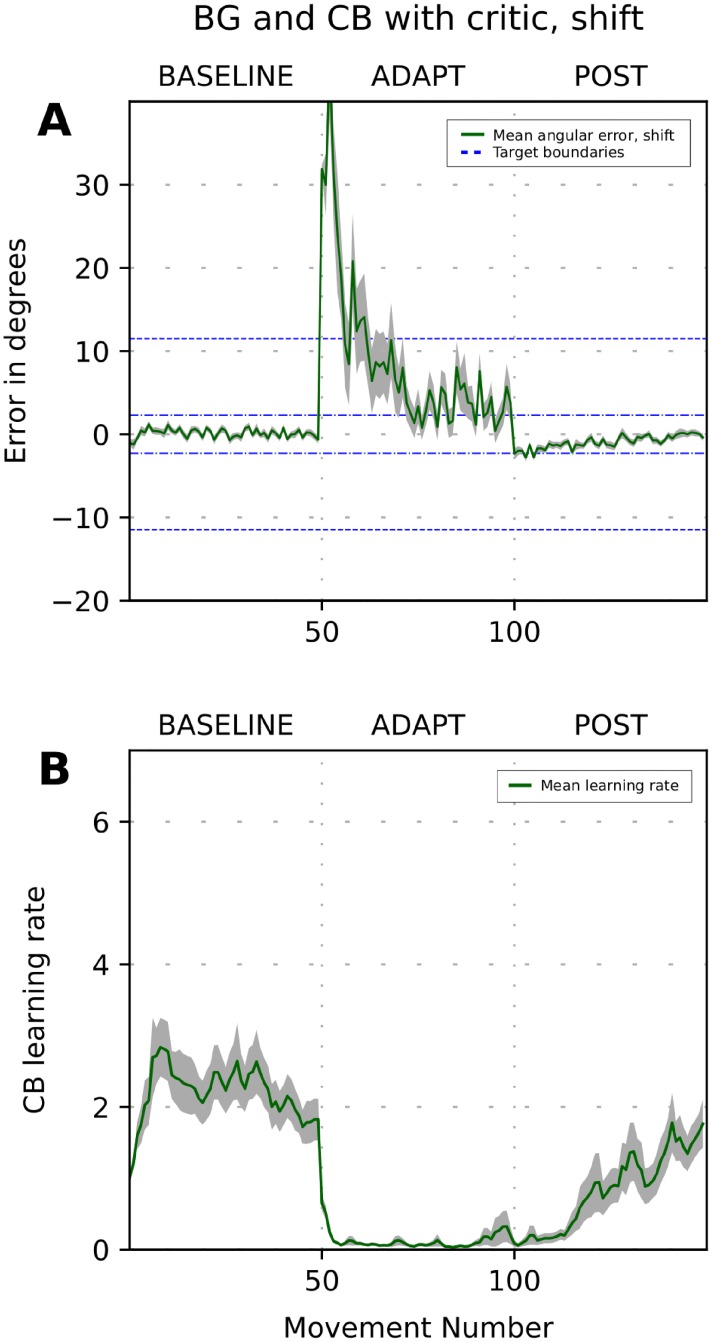
Basal ganglia take over from cerebellum during adaptation to a mild perturbation with the critic-controlled learning rate in cerebellum. The plot shows error magnitude versus trial number during simulation of the 3-phase experiment when a visuomotor perturbation is introduced in the beginning of ADAPT phase and removed in the beginning of POST phase. (A) Solid green line shows 32-run average angle difference between the direction to the target and to the endpoint of the reaching movement; (B) 32-run average CB learning rate dynamics. Dashed lines show the rewarding range, dashed-dotted lines show endpoint noise amplitude. Grey area shows SEMs.

Adding the critic component controlling the learning rate in the cerebellum based on the performance assessment helped to suppress faulty error-based adaptation in case of strong visual perturbations. However, in case of mild perturbations (e.g. a visual shift or rotation by a small angle), when the error-based learning was supposed to be the operating adaptation mechanism, our model failed (see Fig 11 and previous sections). Instead, it exhibited reinforcement-based adaptation (triggered by negative reinforcement) and subsequent exploration when the perturbation was introduced. Therefore, in case when the cerebellar correction is efficient, there should be a mechanism that offsets the effect of negative reinforcement and thus prevents exploration.

Reducing BG-induced errors artificially corresponds to turning off BG learning completely—these are simulations shown on Fig 2. The automatic switch happens with the help of the critic mechanism and is described in subsection “Error-based vs. non-error-based learning and effects of neurodegeneration in BG” below. There the BG involvement is suppressed during adaptation to the perturbation where cerebellum could perform without problems.

We propose that the control signal generated by the critic regulates both supervised learning in the cerebellum and reinforcement learning in BG. In the cerebellum, it defines the learning rate as described above. In BG, it adds to the signal representing the reward prediction error (see Fig 9 and Methods). In this manner, negative RPE resulting from the perturbation combines with a positive input from the critic to form a positive reinforcement signal. This provides an efficient mechanism to disable negative reinforcement and exploration in case when cerebellar correction is adequate. In case when cerebellar correction becomes faulty, the signal from the critic changes to zero, which removes positive offset of the reinforcement signal and thus reenables exploration and reinforcement-based motor adaptation.

We explore the regime where BG and CB work together being regulated by the critic in subsection “Error-based vs. non-error-based learning and effects of neurodegeneration in BG”. We put this part of the manuscript in “Model validation” section because there we reproduce the study [5] in the context similar to the ones presented above.

### Model validation

In this section we test our model performance against literature data that include experimental paradigms combining both error- and non-error-based motor adaptation.

#### Error-based vs. non-error-based learning and effects of neurodegeneration in BG

To study both the error-based and non-error-based motor adaptation and to test how the BG related neurodegenerative diseases, such as Huntington’s (HD) and Parkinson’s (PD) ones, affect the non-error based learning, Gutierrez-Garralda et al. [5] used an experiment during which participants were required to throw balls at a target. During the experiment, either a dove or a wedge prism was used to perturb visual perception of the participants. The dove prism reflected the image about the vertical axis, and wedge prism shifted the visual field horizontally. As mentioned above, the reflection perturbation inverted the sign of the throw mismatch along the horizontal axis, while the shift did not perturb the vector error. Correspondingly, Gutierrez-Garralda et al. assumed that the mechanism of motor adaptation in case of the shift perturbation was an error-based type, while in case of the reflection the participants used a non-error-based motor learning.

Three subject groups participated in this experiment: the healthy control group, the group of patients with Parkinson’s Disease (PD group), and the group with Huntington’s Disease (HD group). All three groups were equally successful in adapting to the shift perturbation (see Fig 4A in [5]) suggesting that PD and HD do not affect error-based learning. However, PD and HD groups were not able to adapt to the reflection as opposed to the control group (Fig 4B in [5]). Therefore, it was proposed that non-error-based motor adaptation is mediated by reinforcement learning in BG, while error-based learning occurs in some other brain structures, presumably in cerebellum [2, 3, 5, 14].

With our model, we simulated an analogous experiment involving reaching movements [8]. In Gutierrez-Garralda et al. experiments, the subjects were not provided with an explicit reward. In our simulations, we assumed that the reward obtained at each trial could be described by a score monotonically decreasing with the error (distance to the target) (see Methods). Therefore, the closer the movement endpoint was to the target, the higher was the reward. PD and HD conditions were simulated in the same way as in [8] (also described in Methods). In short, PD condition involved a reduced rate of reinforcement learning in BG to mimic degeneration of dopaminergic neurons in substantia nigra pars compacta (SNc). Huntington’s disease condition was simulated as a reduction in output of the indirect pathway striatal medium spiny neuron population.

**Fig 12:**
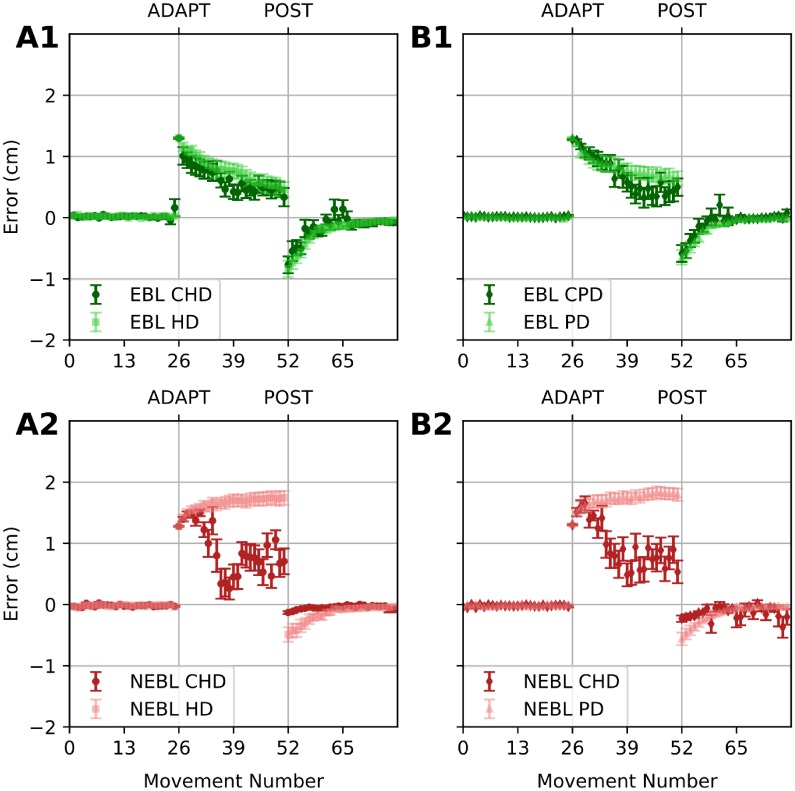
Simulation of error-based and non-error-based adaptation with impaired basal ganglia function. These simulations reproduce Gutierrez-Garralda et al [5] data. (A1, B1) 16-run averaged reaching error for the adaptation to a 30-degree shift perturbation. (A2, B2) 16-run averaged error for the adaptation to an x-reflection perturbation. Shape and darkness of the makers code different conditions: light squares show the simulation of Huntington’s Disease (HD) conditions, and light triangles are used for the simulation of Parkinson’s Disease (PD) conditions. Dark circles and diamonds are used to show control conditions for HD (CHD), and for PD (CPD), respectively. Green color is used for the shift perturbation which engages the error-based learning (EBL). X-reflection perturbation simulation (where non-error-based learning (NEBL) is involved) is shown in red. Error bars show SEMs. Controls reduce the error in both conditions but show almost no aftereffects after x-reflection perturbation. In the shift perturbation simulations (A1, B1), the error reduces equally for both control and PD/HD conditions. After the x-reflection (A2, B2), adaptation occurs in control conditions, but not in PD and HD conditions.

Our simulations shown in Fig 12 are in a good agreement with Gutierrez-Garralda et al. experimental data (Fig 4 in [5]): (1) Simulated adaptation time-course and aftereffects for control and both PD and HD conditions are very similar in case of the visual shift/wedge prism perturbation (Fig 12A); (2) In all conditions there are virtually no aftereffects following the reflection /dove prism perturbation (Fig 12B); (3) In PD and HD conditions, unlike controls, there is no adaptation to the reflection perturbation (Fig 12B).

The mechanistic interpretation provided by the model supports the idea that since visual shift does not perturb the vector of the movement error, all group of participants in [5] successfully used a cerebellar error correction mechanism (error-based learning [5]). After the perturbation was removed, the correction, accumulated in the cerebellum, led to a significant aftereffect (Fig 12A). However, in case of reflection perturbation, the cerebellar learning mechanism became inefficient, and, therefore, suppressed by the critic. The participants switch to reinforcement mechanisms mediated by BG (non-error-based learning [5]). This explains both the lack of the aftereffect and why the adaptation is not occurring in BG-impaired subjects (Fig 12B).

#### Reinforcement-dependent memory retention

In another recent publication by Shmuelof et al. [7], action reinforcement was implicated in motor memory retention. The experimental setup used in that study provides an excellent test for our model. In their experiments, human subjects performed fast reaching movements. They received an explicit reward (via a tone) if they succeeded to reach a target within a predetermined distance (see details in Methods, and in [7]). Most of the time, they also received complete visual feedback provided on a screen.

There were four slightly different versions of the protocol; all of them included 6 phases: (1) during the baseline phase, participants reached to a target for 20 trials; (2) during the adaptation phase, a 30-degree visual rotation was applied to the image on the screen for 60 trials; (3) during the phase they called “asymptote”, for 80 trials the rotation angle remained the same for the participants to better learn this condition (protocols 1 and 2); however, in some experiments the visual feedback was turned off (protocols 3 and 4); (4) with both visual and reward feedback on, participants had to adapt to a larger 45-degree rotation perturbation for 30 trials; (5) in the error clamp phase, during which false visual feedback showed perfect performance, participants were provided with reward and visual feedback; both indicated that the target was successfully reached regardless of the actual arm movement direction either for 60 trials (protocols 1 and 3) or 100 trials (protocols 2 and 4); (6) finally, in the washout phase, all perturbations were removed (as in the baseline phase) for 40 trials.

The major difference between the described experiments was whether the visual feedback was provided in phase (3) or not. Even though there was no significant difference in subject’s performance during this phase (see Fig 2 in [7]), there was a striking difference in behavior during the error-clamp phase (5). Specifically, the subjects that were constantly provided with visual feedback during the asymptote phase (5), were gradually converging their movements to the unperturbed target. In contrast, subjects who were not provided with visual feedback during the asymptote phase, adapted their movements to the target position rotated by 30 degrees (same angle used in the asymptote phase). Therefore, the subjects that did not have visual feedback during 30 degrees rotation, were able to “recall” their motor response to this perturbation during the error-clamp phase. Based on this result, Shmuelof et al. [7] speculated that during the phase when visual feedback was not provided, the subjects followed a different kind of motor learning process (reinforcement learning), suggesting that reinforcement learning was necessary for motor memory retention. Important to note that Shmuelof et al. reported that only 20% of the participants could consciously detect the presence of the rotation perturbation and even less of them noticed the presence of the error clamp.

**Fig 13:**
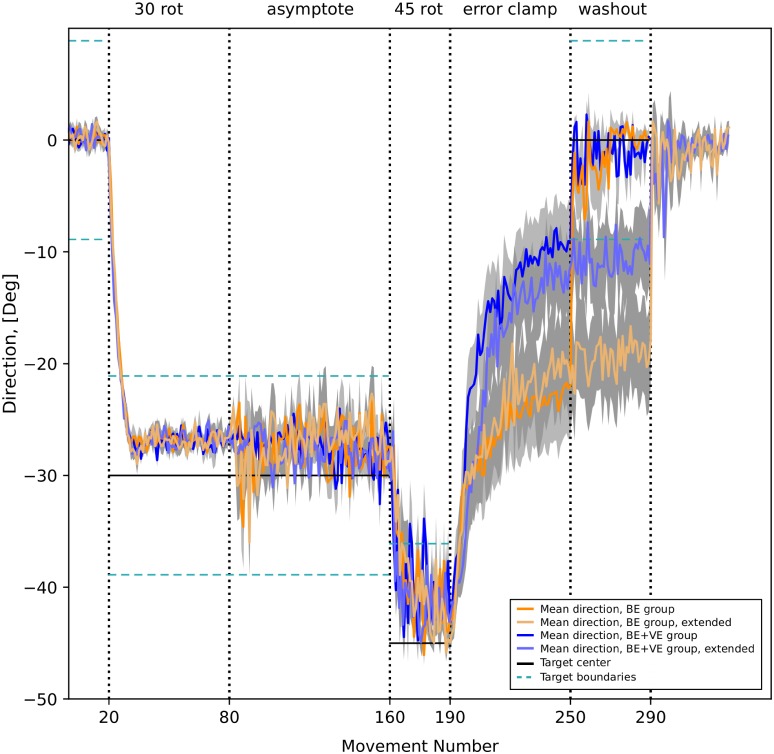
Simulation of the reinforcement-dependent memory retention. These simulations reproduce Shmuelof et al. [7] data. Blue lines show the 16-run average of the reaching angle dynamics for the model that constantly received both binary and vector error feedback (BE+VE). Orange lines show 16-run average of the reaching angle dynamics for the model that received only binary feedback between trials 80 and 160 (BE). Lighter blue and lighter orange lines correspond to the simulations where the error clamp was extended by 40 trials. Thick black horizontal lines show the target center, thin grey horizontal lines show target boundaries. Grey areas show SEMs. During error clamp the orange BE curves (unlike the blue BE+VE curves) approximately converge to the level where the binary feedback only was provided between trials 20 and 160.

Our model reproduces Shmuelof et al. data (compare Fig 13 and 2 in [7]) and provides an interesting mechanistic interpretation of their results. In Fig 13 blue curves show the reaching direction vs. trial number for the group who was continuously provided with both the reward (binary error (BE)) and visual feedback (vector error (VE)). Accordingly, this group is labeled “BE+VE”. Red curves depict the reaching directions for the group who did not have visual feedback during the asymptote phase (between trials 80 and 160). This group data is labeled “BE”. During the simulation, the BE+VE group relied on the cerebellar error-based mechanisms for adaptation exclusively. In contrast, the BE group switched to reinforcement learning during the asymptote phase when no visual feedback was given. So, during the asymptote phase, the BG explored and selected a new rewarding action/motor program. Then, during the 45-degree rotation phase, since visual feedback was provided again, the system switched back to the error-based learning, thus reinforcing the motor program found during the asymptote phase. During the error clamp phase, the degradation of the CB network synaptic weights (the “forgetting” process) gradually diminished cerebellar correction and revealed the unadjusted motor program which appeared different for the two groups. In the BE+VE group, where BG were never involved, this action coincides the default response observed during the baseline phase. In the BE group, however, the motor program was replaced through exploration and reinforcement to the movement in 30-degree direction during the asymptote phase.

#### Learning deficits in cerebellar ataxia and parameters of the critic

Previously we have shown that it is possible to reproduce Schlerf et al [10] data using constant learning rate and increasing the degradation rate for the ataxic condition simulations. Now we show that there is another way of qualitatively reproducing the same data if the learning rate is controlled by the critic. Here we assume that BG are not active, so the critic only controls the learning rate of the CB model.

As explained above, critic decisions are based on whether the difference of the observed and predicted errors falls within a certain threshold, or not. Therefore, by modifying this threshold size, the critic decisions can be modified. We assume that the threshold is proportional to the participant’s estimate of its movement variability, i.e. in control conditions it is equal to the standard deviation of the baseline endpoint noise. Our simulations (not shown) confirm that when the model uses critic thresholds corresponding to underestimated movement variability, it leads to reduced adaptation levels. These simulations support the hypothesis about the misjudged movement variability in cerebellar condition formulated in [10].

For consistency, we also verified that, as in case of the constant learning rate (see *Model Calibration* section), the increased endpoint noise with movement variability estimate used by the critic adjusted correspondingly, does not produce learning deficits. However, if one additionally increases the degradation rate in the cerebellum, the adaptation deteriorates as in Fig 3.

## Discussion

As previously stated, both basal ganglia and cerebellum may be involved in motor adaptation. Although the functional role of each compartment has been investigated, the interplay between the two structures remains poorly understood [15]. We used mathematical modeling to characterize the functional interactions between these structures during motor adaptation.

We introduced a novel model of the network implementing error-based (supervised) learning in cerebellum. Conceptually, this model can be considered a state-space model, like the one discussed in [16]. However, in contrast with more abstract error-based motor adaptation models, our implementation of the cerebellar network is incorporated into the movement system that produces biophysically accurate movements [9]. There are more detailed models of the cerebellum (e.g. [17]), but that was not our goal to model cerebellar networks *per se*. We were rather interested in mechanisms that engage different learning systems depending on context.

In our study, we assume that the role of basal ganglia is to select an action in response to a visual cue. In the context of reaching movements, an action is a temporal pattern of inputs (a motor program) to the motoneurons which activate arm muscles and, thus, generate a movement to a desired target position. We also assume that a copy of the selected motor program is sent to the cerebellum whose role is to calculate and apply a correction to the motor program that reduces the magnitude of the movement error observed on a previous trial. Under these assumptions, we show that if unregulated, action selection and error correction mechanisms come into conflict. We conclude that only one of them should be active at a time and propose a critic mechanism that controls learning rates in both structures. Specifically, the critic predicts the effect of cerebellar correction calculated on the previous trial and compares it with actual movement error perceived by the subject. If the results are consistent, the critic increases its output which defines the learning rate in the cerebellum and increases dopamine release in basal ganglia. If the observed error differs too much from the prediction, the critic decreases its output, which suppresses learning in the cerebellum and reduces dopamine release in basal ganglia. Note, that comparing the magnitudes of the predicted and observed errors is equivalent to comparing predicted change (reduction) of the error between consecutive trials and the observed change of the error. In the context of adaptation to force field perturbations, the idea of variable learning rate, regulated by consistency of the environment has been previously proposed [18]. Our implementation of the critic component follows a somewhat similar idea, though the results are difficult to compare directly because of different experimental protocols being considered.

We show that the suggested critic mechanism provides switching between error-based and non-error-based learning depending on the efficiency of the former. In a nutshell, once the perturbation is introduced, the target is missed resulting in a negative reinforcement (via a reduction of dopamine release) of the movement previously selected by basal ganglia. However, if the correction applied by the cerebellum is successful, the critic facilitates learning in the cerebellum while simultaneously returning dopamine release in basal ganglia to a normal level thus preventing the negative reinforcement of the action from happening. In case the cerebellar correction did not work as planned, the critic stops the learning process in the cerebellum, and allows basal ganglia to block the unsuccessful movement and to initiate exploration of more rewarding actions through the reinforcement learning process.

It is common to classify learning as explicit or implicit [19] and in the context of this study we work under the assumption that all the learning happening in the experiments we reproduce with our model is implicit. I.e. we assume that the feedback manipulations and other experiment conditions (such as high movement speed requirements), used in [5, 7, 10], have prevented cognitive strategies to develop in participants. We stress that PFC in our model PFC node only provides results of direct sensory processing (like CB error and perception of cue activation). It does not play a direct role in the implicit reinforcement learning.

It is certainly likely that true neural mechanisms regulating interaction between supervised and reinforcement learning are more complicated than the ones we present in the paper. However, in the context of “all models are wrong, but some are useful”, we made a simplest possible model that reproduces effects observed in various behavioral experiments. “Simplest” here means within a class of models that follow existing well-established knowledge from electrophysiological and anatomical studies. We have checked several most natural ways of the system organization and ruled out all besides the one with the critic affecting both BG reward and the CB learning rate. Roughly speaking, we show that supervised and reinforcement learning cannot coexist, some switching should happen, provided that one restricts oneself to the classical models of BG and CB like the ones we have used.

We don’t claim that our model is the only one possible, but since to our knowledge it is the first one of this kind, it should be a good start to verify (or falsify) it with more detailed behavioral/electrophysiological studies and create a more accurate model later. We think that currently there is not enough experimental data available to create a plausible model describing more sophisticated interactions between the learning systems. We hope that our model can help to design such experiments.

As noted above, to build our model we had to make a general assumption about the existence of the functional dichotomy between BG and CB. There are, however, recent articles [20, 21] challenging this view, so we stress that this dichotomy is a hypothesis and not a well-established fact. Calcium transients in granule cells have been shown to correlate to reward prediction [20]. Parallel fibers could also be a source of contextual input to the cerebellar nuclei.[21] show that calcium transients in climbing fibers contextually correlate to motor outcomes rather than to motor error. The authors indicate that these results support the hypothesis that climbing fibers provide sensorimotor predictions.

In our model the error is encoded somewhere outside of the circuit. We generally presume that it is communicated to the model directly from the cortex, but for the normal functioning of the model it is not really important which structure supplies the error information. Experimental research has identified that the climbing fibers and the dopamine neurons are strong candidates for encoding errors. We see such data contributing to more accurate description of BG (e.g. [22]) and CB models separately, whereas we tried to use BG and CB models that are as simple as possible and study interactions between them instead.

### Choice of level of detail

Our cerebellum model is essentially a nonlinear version of the standard Kalman filter-based model [10]. And locally it still works in a linear “gradient-descent” way. We have used a nonlinear model instead of a linear one because we wanted to reproduce experiments involving naturalistic complex movements in 2D space. One of the benefits of such approach is the ability to explore effects appearing for strong visuomotor perturbations like rotations by large angles and visual field reflections.

### Adaptation efficiency comparison

Since the critic essentially turns off involvement of either BG or CB in the learning process, the adaptation efficiency of the full model is the same as when the corresponding structure is the only one active from the beginning.

In case when both types of system can lead to successful adaptation reinforcement learning would generally have slower convergence but can potentially stop very close to the target (provided that the rewarded spot it small enough), whereas supervised learning would give fast convergence, but it will stop at the point where degradation (damping/forgetting) in the CB model compensate the learning rate. So in total the answer to the question “Which system adapts better when both potentially can do it?” would depend both on the notion of “better” (e.g. whether one wants fast adaptation or precise adaptation or both) and on a relationship between BG learning rate, reward size, reward spot size, CB learning rate and CB degradation rate.

### Physiological basis for control of motor learning

Although some aspects of cerebellar learning have been characterized, the neurophysiology that underlies cerebellar learning is not fully understood. For instance, while it is known that the CB relies on vector error for learning and that a critic mechanism is necessary for certain motor adaptation tasks, it is not yet clear exactly where or how these mechanisms occur in vivo.

Previous studies suggest that a combination of simple (vector error) and informed (predicted and actual error comparison) feedback is necessary for cerebellar motor adaptation. One potential source of informed feedback is the inferior olive (IO), a nucleus in the medulla, which receives sensory input and provides feedback to the cerebellum via climbing fiber inputs. Gellman et al. [23] demonstrated that subpopulations of IO cells respond to specific sensory modalities. Furthermore in [24], they showed that IO cells were excited in response to unexpected stimuli–e.g. if a participant unexpectedly touched an object during exploration–with subpopulations of olivary neurons responding as sensation-specific “event detectors”. When unexpected stimuli occur, excitation in IO neurons elicits “complex” spikes in cerebellar Purkinje cells, thereby strongly inhibiting neuron activity in the cerebellum. Thus, it is thought that the IO inhibits cerebellar output when reliable sensory information is not available, and therefore the IO likely corresponds to the cerebellum-controlling critic in the model. Anatomical evidence suggests that in order to determine the reliability of sensory input the IO may compare error signals from different sources, though the exact nature of this comparison mechanism is not yet fully understood [25].

Although excitation of IO cells produces complex spikes in Purkinje cells, complex spiking can be caused by other inputs to the cerebellum. In a follow-up study to [24], it was demonstrated that complex spikes could occur in the cerebellar Purkinje cells without the introduction of unexpected stimuli [26]. Thus, there are other sensory inputs, aside from the IO, that cause complex spikes during cerebellum-directed movements; these non-IO inputs likely provide vector error to the cerebellum during normal learning [26].

Another possibility how CB learning rate could be controlled is via some sort of direct control of plasticity in the cerebellum. On the reinforcement learning side the reward modification by critic (which can be a part of IO as noted above) can be done either via modification the signal received by SNc or by direct modulation of dopamine amount in the striatum.

### Choice of model parameter values to simulate different experiments

Our model can be easily modified to produce functional motor adaptation consistent with different experimental protocols. Several related experiments have been selected from the literature allowing for investigation of BG-CB interactions across a range of different motor behaviors. By including a variety of experimental protocols in our simulations, we demonstrated that our model allows generalization across different modalities (e.g. types of movement, implicit/explicit reward, etc.). At the same time, model parameters were kept consistent whenever possible so that simulation results related to different protocols could be compared. Yet, certain parameters were adjusted, such as reward components’ magnitudes, pools of actions used for different tasks, or movement variability as the experiments we model did not have identical experimental settings. We also had to adjust some of the critic parameters. We believe that differences in experimental conditions justify these parameter modifications. In Schlerf et al. experiments [10], the participants made slicing movements wearing a cotton glove (to reduce friction between the hand and table surface) to random targets of 1 cm width within 10 cm from the start location. In Shmuelof et al. study [7], the protocol required subjects to perform fast reaching movements, having arm supported on a lightweight sled that hovered on air cushions created by compressed-air jets (allowing frictionless planar motion of the arm) to 1 cm wide targets 8cm away from the start location. In Gutierrez-Garralda et al. study [5], participants made throwing movements. All these experiments address similar types of motor adaptation and are likely to have similar mechanisms involved, but movement-related and reward-related parameters can vary. A detailed experimental study investigating the validity of our assumptions about the parameters in each particular case is desired, though.

In the current work habit formation (increase of the strength of projections from PFC cue-encoding neurons to the thalamus) does not play a major role, since it is a relatively slow process. As we explored in [8], habituation can play an important role in experiments with larger numbers of trials (and also in the pre-learning phase if it is present) compared to the motor learning experiments we were interested in. Here the numbers of trials were relatively small and the results would be the same if we were to turn off the habit formation in the model completely. We did not do explicit pre-learning of the model in this study and just set “habitual” associations artificially in the beginning. It would not be correct to say, however, that habits themselves don’t play any role at all in the current study, since the number of the trials when BG starts to explore depends on the strength of the initial habitual associations—roughly speaking, the stronger the initial association, the larger the number of punishments from unsuccessful trials that the BG has to receive before it starts to explore.

The model of CB alone has two main internal parameters: (constant) learning rate and degradation rate. There is also an external parameter–noise amplitude. Changing the learning rate controls the speed of adaptation. Also a nonlinear relation between the degradation rate and the learning rate controls the final error level, where learning is balanced by forgetting (changing the degradation rate has a much stronger influence on it). The performance does not drop much with the increase of the noise level.

We studied parameter sensitivity of CB with critic active in the Schlerf et al [10] context (healthy subjects and cerebellar patients adapting to abruptly and gradually introduced rotations). We found that in this case the performance (average size of error) worsens for higher noise levels, but only if it is increased without increasing the model’s estimate of the environmental variability (i.e. if the noise becomes stronger but the model is aware of that, it performs equally well). There is no explicit learning rate, because it is controlled by the critic. There is however the coefficient regulating the maximal learning rate. Increasing degradation reduces the performance in a way similar to the model with a fixed learning rate. There are also parameters regulating the rate at which the critic increases the learning rate if it thinks that everything works fine, and the rate at which it decreases the learning rate when it thinks the opposite. The effects of changing these parameters are the most prominent under the increased noise condition: both reducing the former and increasing the latter (separately) lowers the performance. See Methods subsection “Simulation with critic active” for exact values and more details.

### Error-based adaptation and cerebellar deficits

Via simulation of the experimental results published by Schlerf et al. [10], we demonstrated that our cerebellar correction model replicates motor adaptation data to single step and multi-step visual rotations. We also reproduced adaptation deficiencies observed in ataxia patients by increasing the degradation rate of synaptic weights in the cerebellar network to represent a reduction in memory related coefficient inferred by Schlerf et al. [10] using a Kalman filter model.

Interestingly, Schlerf et al. have not found any significant alterations in the learning rate related parameter of their model in ataxia patients, even though their motor variability was increased. This is consistent with Butcher et al. results [27], where it was shown that increased movement noise alone does not lead to deficits in adaptation. Our model also shows no change in the cerebellar learning rate if an increase in movement variability is accompanied by the proportional increase of the thresholds used by the critic. In fact, instead of increasing degradation, one could reduce the learning rate (though the amount of required change is higher) to produce similar changes in the adaptation levels. However in this case these adaptation levels (asymptotic error values) would be reached significantly slower which was not observed in the behavioral data by Schlerf et al. [10].

Integration of the critic into the model provides additional possibilities to interpret Schlerf et al. [10] results. Our simulations show that similar learning deficits appear if one changes some of the critic parameters. In particular, the increase in the asymptotic error in multistep condition can be obtained by reducing the movement variability estimate used by the critic to evaluate the efficiency of the cerebellar correction (or increasing the endpoint noise level without adjusting the parameters of the critic). It goes in agreement with Schlerf et al. hypothesis [10] that ataxics have a wrong estimate of their own movement variability. Similar effects were observed when the reaction of the critic to the threshold crossing was modified. However, the changes of these critic parameters led to a much more dramatic reduction of the learning rate, than the increase of degradation.

In Schlerf et al. analysis [10] the learning rate of control participants was greater after the single step perturbation, than after the multi-step more gradual perturbation. In our simulations we did not observe this effect. In our simulations the difference of the learning rate in controls in multistep and single step conditions observed for some parameter values happens because in multistep condition unexpected effects (noise and perturbations) are smaller on average, than in a single step condition, thus the critic is less likely to reduce the learning rate. In a broader context, in our model both underestimating and overestimating the uncontrollable movement variability by the critic can have negative effects on learning. Underestimated variability can lead to overly strong reduction in the learning rate for mild perturbations, which results in reduced ability to adapt using error-based learning and may trigger BG-driven exploratory behavior. Overestimated variability tends to drive cerebellar learning rates close to maximum possible and prevent the system from switching to non-error-based learning mechanisms, if it becomes necessary.

### Predictions

The model has implications that can be tested experimentally. It predicts, for example, that for very precise movements, where the expected movement variability is low, the participants are less likely to adapt even to mild perturbations, in comparison to the case when their movement variability is artificially increased. The good test for the model would be a Gutierrez-Garralda-like experiment with reaching movements under the rotation perturbation instead of the Dove prism and with explicit rewards. It would be important to check whether the observed effects qualitatively change for different movement variability magnitudes. Another way to test the model is to perform a series of experiments with different visuomotor rotation angles and check at what angles participants switch from error-based to non-error-based learning.

Another important prediction of the model is variations of the striatal dopamine concentrations. Specifically, our model predicts that during error-based adaptation the dopamine levels are significantly higher compared to the non-error-based scenario. To test this prediction, one could measure the striatal dopamine concentration during adaptation to visuomotor perturbations implying different (error-based vs. non-error-based) learning mechanisms.

## Conclusions

The proposed model of motor adaptation to perceptual perturbations includes two distinct learning structures, cerebellum and basal ganglia, responsible for error-based and non-error-based motor learning, respectively. We demonstrate that based on existing experimental evidence, it is necessary to have a mechanism regulating involvement of cerebellum and basal ganglia depending on the perturbation. We suggest that the involvement of a particular learning mechanism is regulated by the same signal depending on the consistency of the internal model used by the brain to predict the movement results. The resulting model reproduces data from several experiments, involving interaction between error-based and non-error-based learning mechanisms.

## Methods

We distinguish between the model of the motor adaptation system, which is supposed to reproduce activity of some parts of the participant’s brain, and the model of the experimental environment, which is supposed to describe factors and events that the participant does not control. We call the latter “a context”.

The model aims at representing a human subject performing reaching movements with indirect visual feedback of her/his arm during a sequence of trials. We assume that the hand position is not directly visible but is displayed on a screen as a cursor. At the beginning of each trial the participant places his hand to a fixed starting point and the attempts to move the arm to a target position appearing on the screen 20 cm away from the starting position. The visual feedback (hand position-cursor position correspondence) can be artificially perturbed during the experiment. Depending on the context, the participant receives a reward if the movement endpoint occurs within the target spot.

We used different models of the motor adaptation system of increasing complexity and different contexts. Each context corresponded to one of the experimental protocols, which included experiment-specific alterations of the visual feedback and reward paradigms.

### Neuro-mechanical model of the arm

To simulate center-out reaching tasks, we previously designed an arm model simulating 2D center-out reaching movements described in details in [9]. In short, the model consists of two rigid links connected by hinge joints (shoulder and elbow) actuated by six Hill-type muscles [28]. These include four single-joint muscles: the shoulder flexor (SF) and extensor (SE), the elbow flexor (EF) and extensor (EE), which control rotation of either the upper arm or forearm around the corresponding joint. The other two muscles, namely: bi-articular flexor (BF) and extensor (BE) are two-joint muscles that attached to both joints and simultaneously control movement around them. The arm movement depends on the combination of multiple muscle activations and is restricted to the horizontal plane. The dynamics of the arm motion is derived from the Lagrange equations, which take into account the Coriolis and centrifugal forces, joint viscoelastic forces, and muscle forces [9]. The proprioceptor afferent feedback from each muscle (Ia and Ib) projecting to the spinal cord was derived and modified from [29].

The model of spinal cord comprises complex interconnections among motorneurons, interneurons, including Renshaw cells, Ia- and Ib- inhibitory interneurons and correspondent afferent feedbacks (see [9] for details). The spinal circuitry receives descending inputs from the motor cortex that activates corresponding motoneurons, which in turn drive arm movements via activation of muscles. Interneurons and Renshaw cells mediate interactions within spinal circuits and modulate cortical signals to the arm muscles. In addition, spinal reflexes, namely: stretch reflex, autogenic inhibition reflex and recurrent inhibition of motoneurons, which play an important role in arm kinematics [30], are incorporated into the model of spinal cord. The detailed description of the spinal circuitry can be found in previous publications [9, 30, 31].

The position and movement of the arm is controlled by the time varying input descending from cortex which we refer to as a motor program. So, the motor program is a time-dependent 6-dimensional vector of signals innervating the spinal cord circuitry, ***p***(*t*). To actuate reaching movements, we implemented cortical neurons as a cortical "controller" that solves an inverse problem

$$F(p,0)={\vec{x}}_{0}$$

where ${\vec{x}}_{0}$ is the target position. The inverse problem solution is based on a proposed straight-line trajectory to a target position and a predefined bell-shaped arm endpoint velocity profile. Thus, the controller generates a motor program that produces a task-specific activation of low-level spinal circuits that in turn induce the muscle activation pattern realizing the intended reaching movement. Therefore, calculation of the correct motor program relies on the information about target position which in turn depends on how reliable visual feedback is. During visual perturbations, the target position is perceived incorrectly, which thus results in a displacement of the movement endpoint relative to the actual target position, i.e. in a movement error.

### Model of cerebellum

The role of cerebellum in the model is to calculate a correction to the cortical motor program that brings the movement endpoint closer to the target position. This correction is assumed to be additive and is calculated based on the information about the movement error acquired on the previous trial. The corrected motor program ***p***_*cor*_(*t*) is calculated by linearly transforming the original motor program

$$p_{cor}(t)=(1+W)p(t)$$

where *W* is a 6×6 correction matrix. This correction can be viewed as a result of processing the initial motor program by a linear artificial neural network mapping 6 inputs to 6 outputs with synaptic weights *W*. Weight matrix update follows classical error back propagation algorithm which is a one-step iteration of the gradient descent method to minimize the magnitude of the vector movement error $\vec{e}$:

$$W_{new}=W-\frac{1}{2}\lambda \frac{\partial }{\partial W}{\vec{e}}^{T}\vec{e}-\gamma W$$

where parameter *λ* defines the convergence speed, and *γ* defines exponential decay of the synaptic weights in absence of learning signal (forgetting). Hereinafter we refer to *λ* and *γ* as learning and degradation rates, respectively.

Taking into account that the movement error is the vector difference between the movement endpoint position and the target position ${\vec{x}}_{0}$, we have

$$\vec{e}=F(p,W)-{\vec{x}}_{0}$$

where *F*(***p***, *W*) ≔ *F*(***p***_*cor*_, 0) is the movement endpoint as a functional of the initial motor program ***p***(*t*) and the correction matrix *W* as calculated by the neuro-mechanical arm model with the corrected motor program. Then the equation for the weights update takes the form

$$W_{new}=W-\lambda \frac{\partial F}{\partial W}\vec{e}-\gamma W$$

where *∂F*/*∂W* is a 6×6×2 tensor whose components are the derivatives of the end movement positions with respect to correction matrix entries.

### Basal ganglia model

The model of reinforcement learning in basal ganglia we used in this study was previously published and is described in details in [8]. Briefly, the model is an extension of the classical two-pathway BG model from [32] to the case of many possible actions. Here, we only provide short qualitative description of our model. Behavioral experiments studying reinforcement learning mechanisms assume that a choice must be made between several differentially rewarding behavioral options. Unlike decision-making tasks, motor learning does not imply a small or finite number of possible choices. The only constraint is the context of the task, e.g. reaching from a fixed initial position to an unknown destination. Our model has unlimited number of possible actions. As the context, we used center-out reaching movements performed in a horizontal plane. To calculate cortical activity corresponding to different movements, we explicitly solved an inverse problem based on the given arm dynamics as described above. Accordingly, for every possible reaching movement we could calculate the corresponding motor program represented by the activity profiles of cortical inputs responsible for activation of different muscles. To describe different experiments, we define corresponding (arbitrarily large) sets of motor programs that define all possible behavioral choices (actions) in each experimental context.

The classical view of action selection is that different motor actions are gated by thalamocortical relay neurons. In the presented model, we assume that relay neurons can be activated at different firing rates, and their firing rates define contributions of different motor programs to the resulting motor response. More specifically, in our model cortical input to the spinal network is implemented as a linear combination of all possible motor programs in the given context with coefficients defined by the firing rates of corresponding thalamocortical relay neurons. This linear combination can be viewed as an aggregate input to the spinal network from the cortical motoneurons exhibiting activity profiles corresponding to different motor behaviors, e.g. reaching movements in different directions.

The classical concept of BG function is that the BG network performs behavioral choice that maximizes reward. This action selection process results in activation of thalamic relay neurons corresponding to the selected action and suppression of neurons gating other behaviors. Per this concept, each action is dedicated to specific neurons in different BG nuclei. Their focused interconnections form action-related loops which start at the cortex, bifurcate in the striatum into direct and indirect pathways converging on the internal Globus Pallidus (GPi), and feed back to the cortex through the thalamus. Action preference is facilitated by increased excitatory projections from sensory cortical neurons representing the stimulus to direct pathway striatal neurons (D1 MSNs). Suppression of unwanted competing actions is assumed to occur because of lateral inhibition among the loops at some level of the network in a winner-takes-all manner.

The classical model predicts that novel cue-action associations acquired based on reinforcement learning rely on BG network integrity. However, multiple experimental studies have shown that pharmacological blockade of GPi, the BG output structure, does not lead to significant impairments in performing well-learned tasks. Consistent with these experiments, it was suggested that acquired associations may become “direct” projections within the cortex bypassing the BG network. In our implementation of the model, competing actions are suppressed by lateral inhibition in the population representing thalamocortical neurons, independent of BG network integrity.

In the model, novel cue-action associations are formed based on reinforcement learning in the striatum. Eventually, the preferable behavior is reliably selected due to potentiated projections from the neurons in prefrontal cortex (PFC), activated by the provided stimulus, to D1 MSNs, corresponding to the preferred behavior. On a longer timescale, repetitive execution of the same action in response to the same stimulus leads to habituation of the response via long-term potentiation of the direct projections between the corresponding PFC and thalamocortical relay neurons based on Hebbian learning. At the same time, due to degradation of synaptic connections between the PFC and striatum in absence of reinforcement, BG involvement in the action selection process gradually decreases. Eventually, the behavior becomes a habit, which is automatically selected solely based on direct cortico-cortical projections.

In technical terms, the output of basal ganglia model is the activation levels of thalamocortical relay neurons in response to the input from PFC neurons activated by visual cues. Each cure represents one of the possible reaching targets. These levels are used as coefficients of the linear combination of all possible actions which represents the motor program selected for execution. The resulting motor program is used to calculate the endpoint of the movement using **Neuro-Mechanical Arm Model** (see above). Depending on the distance between the movement endpoint and the target position, the reward is calculated as dictated by the experimental context (see below). This reward value is used to calculate the reward prediction error (RPE) as a temporal difference between the current and previous reward values. The RPE is used as the reinforcement signal to potentiate or depress synaptic projections from PFC neurons, activated by the visual cue provided, to the striatal neurons, representing the selected actions.

Mathematically, the model is implemented as a 700-dimensional hybrid dynamical system made of distinct 7 populations of rate neurons (D1 MSN, D2 MSN, GPe, STN, GPi, thalamus), each containing 100 rate neurons (each representing a neuronal subpopulation); every neuron corresponds to a one of the parallel loops within basal ganglia-thalamocortical circuit. The parallel structure is only violated in the thalamus node where each loop inhibits all other loops, creating a “winner takes all effect” which prevents strong simultaneous activation of different loops. The simulation of a single trial goes as follows. First, the rate differential equations are solved until a point attractor is reached (which empirically always happens with this system). This point attractor describes activities of all the 700 rate neurons, including the ones in the thalamus. We then take the activities of the thalamic neurons and use them as input to the neuro-mechanical arm model, which in turn produced a movement. After that the reward is supplied (or not) to the model, depending on whether the movement endpoint was in the proximity of the target center (target size is a model parameter). Using the reward value the model computes the RPE and updates the reward prediction value. Finally, the RPE value is used to update to strengths of connections between the PFC and D1 MSN, D2 MSN. PFC neurons (whose number is equal to the number of cues in the experimental setup) are not simulated and are modeled simply by real numbers meaning that they provide constant signal while BG reaches a decision.

See details in [8].

### Model of the Critic

In the presence of visual perturbation, cerebellum may produce corrections that do not lead to improvement in movement accuracy. To cope with that, the critic in the model predicts the result of the implemented correction and compares it with the actual improvement in performance on the next trial. To calculate the expected error after correction, the critic uses the internal model

$${\vec{e}}_{exp}=F(p,W_{new})-{\vec{x}}_{0}$$

where *W*_*new*_ is an adjusted correction matrix as described above, and ${\vec{x}}_{0}$ is the actual target position.

The critic compares the expected error with the error ${\vec{e}}_{real}$ observed on the next trial which is subject to possible visual perturbation. If the observed error agrees well with critic’s prediction, then the critic concludes that CB is functioning correctly and increases the learning rate *λ* in the cerebellum for future adjustments. If there is no agreement, the critic assumes that either the cerebellar correction was not the main reason for the error change (e.g. the altered motor program), or CB was relying on the distorted error when adjusting the correction matrix. In both cases, the critic decreases the learning rate to suppress faulty cerebellar learning. Specifically, the critic calculates the magnitude of the difference between the errors

$$mismatch=|{\vec{e}}_{real}-{\vec{e}}_{exp}|,$$

and then forms its output

$$critic=\left\{ \begin{array}{c} speed_{up}\cdot \lambda ,\;mismatch<t_{low}\cdot \kappa ;\\ \;\lambda ,\;t_{low}\cdot \kappa \leq mismatch\leq t_{high}\cdot \kappa ;\\ \frac{\lambda }{{slow}_{down}},\;mismatch\geq t_{high}\cdot \kappa .\; \end{array}\right.$$

Here the critic compares the mismatch between predicted and perceived errors with two threshold values. These thresholds are multiples of the estimate of the movement variability *κ* which is assumed to be equal to the standard deviation of the endpoint noise. The critic treats any mismatch less than *t*_*low*_ times the endpoint variability estimate *κ* as consistent with prediction, any mismatch greater than *t*_*high*_⋅*κ* as inconsistent with prediction, and any mismatch in between these threshold is treated as inconclusive. The critic output is used to set as a new value for the learning rate *λ* for the next trial. Therefore, in case of the consistent prediction it is increased by a factor of *speed*_*up*_, in case of the inconsistent prediction it is reduced by a factor of *slow*_*down*_, or remains unchanged if the test is inconclusive. The values of the parameters used are specified in each simulation context.

We bound the critic output by 0.001 from below and by *a*_*opt*_ ⋅ *λ*_*opt*_ from above, where ⋅*λ*_*opt*_ is defined as the value of *λ* that leads to a complete elimination of the vector error in one step (${\vec{e}}_{exp}\;=\;0$) in absence of any visual perturbation. Also, if the current error magnitude is extremely small (less than 1mm) the critic signal is accepted equal to current value of *λ*, i.e. the learning rate does not change.

The critic signal is also used to offset the reinforcement signal *DA* in the basal ganglia mode:

DA=RPE+α⋅critic

where *RPE* is the reward prediction error, and coefficient *α* depends on the context (see below). In case cerebellar corrections are efficient in reducing the movement error, the critic output is close to its maximum, which ensures positive reinforcement of the currently selected action regardless of the reward provided. When cerebellar corrections are faulty, the critic output is close to zero, which suppresses learning in cerebellum and creates prerequisites for the reward-based learning in basal ganglia.

### Simulation algorithm

The general flow of information in the model is as follows. First, basal ganglia select a motor program to be executed. Then cerebellum modifies the program and sends it as an input to the neuro-mechanical model of the arm which executes the program and calculates the movement endpoint which is used to calculate the vector error and the reward. Because of visual perturbation, the perceived vector error may be distorted depending on the experimental context. The critic evaluates whether cerebellum correction worked as expected and sets its output accordingly. Then basal ganglia module updates synaptic weights of the projections from PFC to striatal neurons based on the reward received and the critic input, and cerebellum updates the correction matrix based on the vector error received and the learning rate set by the critic.

More formally, the simulation of each trial can be divided into following stages, some of which can be skipped depending on the particular model type (see text and Fig 9B):

1. Selection of the motor program by the BG model, based on presented cue
2. Application of the CB correction using current CB correction matrix
3. Use of the resulting motor program as an input to the arm model to simulate the movement and calculate reaching endpoint
4. Addition of Gaussian (with mean zero and standard deviation 0.005 *m* = 5 *mm* noise to the endpoint) and application of the visual perturbation that transforms the vector error
5. Critic evaluation of the predicted and perceived error agreement
6. Calculation of the reinforcement signal as a sum of the reward and the critic signal
7. BG learning: use of the reinforcement signal to update the synaptic weights of PFC projections to the striatum
8. CB learning: Update of the correction matrix based on the vector error, and the critic signal as a learning rate.

### Simulation contexts (models of experimental protocols)

Description of each of context includes the list of targets and (initial) cue-action associations used, sequence of cue activations, reward description as well as some additional minor details.

#### Test 3-phase contexts

First, we describe simple contexts that we use to demonstrate model performance using progressively augmented architecture (Figs 2, 4, 7, 8, 11 and 12). In total we use three sensory cues (C1 and C2, C2’) and three actions (A1 and A2, A2’) which corresponded to reaching toward North-East (T1, 45 degrees), North-North-East (T2, 75 degrees) and North-West (T2’,135 degrees) targets at 20 cm distance from the starting point. We set habitual associations C1->A1, C2->A2 and C2’->A2’ represented by direct projections from PFC neurons (cues) to thalamocortical relay neurons (actions) to be of strength 0.3 (see [8] for details). In each context we use only two cues and two actions. Then the virtual experiment has the following protocol:

1. Baseline: for 50 trials of the simulation, cue C1 is activated.
2. Adaptation: for 50 (or 100) trials, a visual perturbation is applied.
3. Post-effect: Finally, for the last 50 trials the conditions were returned to the ones used during first 50 trials

The visual perturbation is fixed for every context and can be of the following types: 1) shift perturbation in reaching context: a switch of the presented cue from C1 to C2 (or C2’) while keeping the vector error unaltered directly. 2) Rotation perturbation: a switch of the presented cue from C1 to C2 (or C2’) and CCW rotation by 30 (or 90) degrees is applied to the movement endpoint (hence also to the vector error). 3) X-reflection perturbation: a switch of the presented cue from C1 to C2 and x-coordinate reversal in the movement endpoint (which, in turn, affects the vector error in the similar manner).

We label the protocols in the following way.

- Mild shift context: 3-phase context with 30 degrees shift perturbation during 50 trials;
- Mild rotation context: 3-phase context with 30 degrees rotation during 50 trials;
- Strong shift context: 3-phase context with 90 degrees shift perturbation during 100 trials;
- Strong rotation context: 3-phase context with 90 degrees rotation during 100 trials;
- Reflection context: 3-phase context with reflection perturbation during 100 trials.

For all these contexts, the pool of possible actions consisted of 100 reaching movements with endpoints uniformly distributed from 20 to 155 degrees on a circle with 20 cm radius and a centre at the initial hand position. When the reinforcement learning was enabled, the reward of magnitude 3 was given for reaching within a circular target spot of 4 cm size, centered at the target. The initially reward was set to 3 as well, assuming that subjects were pretrained to perform reaching movements and getting reward of this particular magnitude for reaching within the target spot. For strong shift, strong rotation and reflection perturbations we used the cerebellar learning rate = 2.

#### Schlerf et al. context [10]

The context uses 13 cues with habitual associations with movement to directions from 60 to 90 degrees with a 2.5-degree step, to represent different targets used in the actual experiment. At each trial one of these cues was randomly activated. To simulate trials without visual feedback we did not update the CB correction matrix after them.

The endpoint noise had *SD* = 1.05 cm. For the controls the learning rate was set to 1. The pool of actions was 100 reaching movements in directions uniformly distributed from 58 to 92 degrees.

To replicate the altered adaptation observed in ataxic patients, we increased the degradation coefficient *γ* in the CB model from 0.04 to 0.16 to replicate the experimentally observed asymptotic error. The arm endpoint noise SD was increased to 0.013 to replicate the larger movement variability observed in cerebellar patients.

#### Simulation with critic active

For the version of the context with critic active, the critic thresholds were *t*_*low*_ = 2, *t*_*high*_ = 3.5 and the speeds *speed*_*up*_ = *slow*_*down*_ = 1.3. The optimality coefficient was *a*_*opt*_ = 0.2, to limit the learning rate values–for this setup due to constantly changing targets and large noise the learning rates tend to get high values for our critic parameters. The movement variability estimate was equal to the noise SD.

We have used several modifications of parameters for controls, which can be regarded as a study of how critic performance depends on its parameters. Increasing both noise to 0.013 and estimate of the variability to 0.013 does not lead to any change both in rates and in adaptation levels. If we additionally increase degradation to 0.016 (like in the constant rate case), we reduce the adaptation level, but the rates stay the same. In fact, for our parameter values increasing noise to 0.013 without adjusting movement variability does not have a strong effect. Though if we do the same, while decreasing the estimate of the variability, the effect is significant. The same happens if we apply stronger noise (not shown). Keeping the noise size at controls level, one can decrease the adaptation levels by reducing the optimality coefficient to 0.05 or the movement variability estimate alone to 0.88 cm. In general, increasing noise and decreasing the movement variability estimate has the same qualitative effect (one can get a quantitative version of it too). The fact that increasing the noise to 0.013 did not produce a strong effect only reflects the fact that we increased the noise not strongly enough to match the decrease of the movement variability estimate to 0.88 cm (which can be translated into sizes of thresholds *t*_*low*_ and *t*_*high*_). For the control noise value changing *speed*_*up*_ and *slow*_*down*_ within large ranges did not produce almost any effect, but for the increased level of noise (without movement variability adjustment) one can get reduced adaptation levels by either reducing the *speed*_*up*_ to 1.12, or increasing the *slow*_*down*_ to 1.7.

Like in the constant rate case, the adaptation levels the model produce are rather stable to the learning rate amplitude alterations (the most direct analog of the strongly reduced constant learning rate is strongly reduced optimality coefficient), but final adaptation level takes longer to achieve for smaller rates. Not surprisingly, unlike degradation increase, critic parameter modifications affect adaptation to single step perturbations stronger than multi-step adaptation, because the model encounters stronger perceptual perturbations. Even though during the multi-step perturbation the perturbations reaches the same magnitude, the formula for the critic we use works with changes of the error rather than with the error itself, otherwise it would not be able to adapt to perturbations strong in magnitude, but not changing perception (e.g. large shifts).

#### Gutierrez-Garralda at al. context [5]

We used two sensory cues (C1 and C2) corresponding to actions A1, A2 of reaching to targets T1 at 70 degrees and T2 at 110 degrees, respectively.

We set the habitual associations for both cues (C1-A1 and C2-A2) represented by connections from corresponding PFC neurons to thalamocortical relay neurons with initial strength 0.55 (see [8] for details). During the simulation, the target T1 corresponded to the actual target position, and the target T2 corresponds to the distorted target position as perceived during the visual perturbation.

For the first 25 trials, the cue C1 was activated, and reward was provided for reaching to the target T1. Then for trials 26 to 50, the activated cue was changed to C2, but the reward was still provided for reaching to the target T1. Finally, in the last 25 trials the activated cue was changed back to C1, and reward was provided for reaching to the target T1 again. The rewarding spot radius (*D*_*max*_) was 4 cm. The pool of actions consisted of 100 reaching movements in directions uniformly distributed from 10 to 170 degrees. The reward value was set to be equal to

$$R=-22{\left|\vec{e}\right|}^{1.5}+0.7\lambda$$

The initial reward expectation for both cues was set to 0.

In the experiment [5] the participants were aware of the perturbation. Therefore, we reset the error history when we introduced or removed the perturbation (i.e. the critic did not update its output on a trial immediately following the change in conditions).

In this simulation context we used the following parameters for the critic component: *t*_*low*_ = 1.9, *t*_*high*_ = 2.1, movement endpoint variability estimate *κ* = 0.005, the learning rate update parameters *speed*_*up*_ = 2 and *slow*_*down*_ = 2.8, and *a*_*opt*_ = 0.8.

Parkinson’s (PD) and Huntington’s Disease (HD) conditions were simulated as described in [8]. Specifically, PD condition involved a 90% reduction in the rates of reinforcement learning in BG to mimic degeneration of dopaminergic neurons in SNc. HD condition was simulated as a 90% reduction in output of the indirect pathway striatal medium spiny neuron population.

#### Shmuelof et al. context [7]

We have used a single cue C1 associated with reaching movement to the North (90 degrees, see Fig 1B). The initial strength of the connection from the PFC neuron representing this cue to the thalamocortical neuron corresponding to this action was set to 0.1.

During the asymptote phase, to imitate absent visual feedback, we set the critic output to zero. We simulated the error clamp phase by setting the vector error to zero and providing the reward regardless of whether the target spot was reached or not. The pool of possible actions was 100 reaching movements in directions uniformly distributed from 40 to 110 degrees.

The participants have received explicit reward in [7], so we modelled the reward in the following way:

$$R=2\Theta (0.028-|\vec{e}|)+0.65\cdot \lambda$$

where Θ(.) is Heaviside step function. The endpoint noise standard deviation was 0.008m. The critic component parameters were *t*_*low*_ = 2, *t*_*high*_ = 3.5, the movement variability *κ* = 0.008, *a*_*opt*_ = 0.2, the rate update factors *speed*_*up*_ = 1.3 and *slow*_*down*_ = 1.3.
